# Cholesterol-dependent cytolysins impair pro-inflammatory macrophage responses

**DOI:** 10.1038/s41598-018-24955-2

**Published:** 2018-04-24

**Authors:** Pushpak Bhattacharjee, Peter A. Keyel

**Affiliations:** 1 Department of Biological Sciences, Texas Tech University, Lubbock, TX 79409 USA; 2 Present Address: Department of Cell Biology and Biochemistry, Texas Tech University Health Science Center, Lubbock, TX 79430 USA

## Abstract

Necrotizing soft tissue infections are lethal polymicrobial infections. Two key microbes that cause necrotizing soft tissue infections are *Streptococcus pyogenes* and *Clostridium perfringens*. These pathogens evade innate immunity using multiple virulence factors, including cholesterol-dependent cytolysins (CDCs). CDCs are resisted by mammalian cells through the sequestration and shedding of pores during intrinsic membrane repair. One hypothesis is that vesicle shedding promotes immune evasion by concomitantly eliminating key signaling proteins present in cholesterol-rich microdomains. To test this hypothesis, murine macrophages were challenged with sublytic CDC doses. CDCs suppressed LPS or IFNγ-stimulated TNFα production and CD69 and CD86 surface expression. This suppression was cell intrinsic. Two membrane repair pathways, patch repair and intrinsic repair, might mediate TNFα suppression. However, patch repair did not correlate with TNFα suppression. Intrinsic repair partially contributed to macrophage dysfunction because TLR4 and the IFNγR were partially shed following CDC challenge. Intrinsic repair was not sufficient for suppression, because pore formation was also required. These findings suggest that even when CDCs fail to kill cells, they may impair innate immune signaling responses dependent on cholesterol-rich microdomains. This is one potential mechanism to explain the lethality of *S. pyogenes* and *C. perfringens* during necrotizing soft tissue infections.

## Introduction

Necrotizing soft tissue infections (NSTIs) are a group of related, potentially fatal bacterial infections, like necrotizing fasciitis and gas gangrene, that quickly spread through and kill soft tissues in the body. The median mortality for NSTI is 32% and approaches 100% without treatment^1,2^. NSTI is often polymicrobial, with both Gram positive and negative organisms present^1,2^. The most frequent solitary pathogens are beta-hemolytic Streptococci or Clostridia, like *Streptococcus pyogenes* (Group A Streptococcus) and *Clostridium perfringens*^1–3^. Both pathogens secrete many virulence factors, including the phospholipase C/sphingomyelinase α-toxin and the pore-forming toxin Perfringolysin O (PFO) in *C. perfringens*^4–8^, and the NAD^+^-glycohydrolase Spn and the pore-forming toxin Streptolysin O (SLO) in *S. pyogenes*^9–13^. Both PFO and SLO are cholesterol-dependent cytolysins (CDCs). CDCs are pore-forming toxins that require cholesterol for binding to the cell membrane^14^. Following binding, CDCs oligomerize into a pre-pore, which then inserts into the cell membrane^14^. Oligomerization and pore-formation can be arrested through the use of well-characterized toxin mutants that generate either oligomerization-defective, nontoxic “monomer-locked” proteins or nontoxic linear oligomeric “array-locked” proteins^15–20^. These mutant toxins are useful tools to uncover the contributions binding and oligomerization play in toxin activity^15,16^. However, the relative contribution of binding and oligomerization to immune evasion by CDCs has not been determined.

CDCs promote immune evasion through multiple mechanisms. One mechanism of CDC-mediated immune evasion is direct killing of immune cells^21–24^. However, CDCs also promote immune evasion nonlethally. CDCs reduce phagocytosis and promote phagolysosomal escape^23,25–28^. SLO can impair the oxidative burst and activation in neutrophils^29^, though the mechanism of impairment is not known. PFO induces vascular leukostasis, which blocks immune cell recruitment to the site of infection during gas gangrene^4–8^. In contrast, during necrotizing fasciitis, leukocytes infiltrate, but do not clear the bacteria^30,31^. Thus, CDCs use several mechanisms beyond cell death to blunt the host innate immune response.

The host’s innate immune response is critical to promoting survival during NSTI because the disease spreads rapidly^8,31^. *S. pyogenes* is controlled by macrophages, which eliminate bacteria and secrete pro-inflammatory cytokines, including Tumor Necrosis Factor α (TNFα)^32^. TNFα production is induced by a wide variety of signaling pathways, including pro-inflammatory cytokines like TNFα itself and Interferon γ (IFNγ), and pattern-recognition receptors such as Toll-like receptors (TLRs)^33–35^. Following TLR activation, the adaptor proteins MyD88 and/or Trif are recruited to the TLR, where they mediate downstream TLR signaling. TLR signaling induces pro-inflammatory cytokine expression and increases cell surface expression of both activation markers like CD69^36^, and costimulatory proteins like CD80, CD83 and CD86^35,37^. Ligation of TLRs also induces the priming of the inflammasome. The inflammasome is a multiprotein complex that senses a wide variety of danger signals. It is comprised of a sensory Nod-like Receptor (NLR), the adaptor Pycard, and an inflammatory Caspase (Casp)^38,39^. The best studied inflammasome, the NLRP3 inflammasome, senses membrane damage, like that caused by CDCs^15,40,41^. Following activation of the sensory NLR, NLRP3, Casp1 is activated, leading to pro-inflammatory IL-1β and IL-18 secretion and the programmed cell death pathway termed pyroptosis^38,39,42^. Pyroptosis is the inflammatory lysis of cells by Casp1 or Casp11 mediated cleavage of Gasdermin D^42-44^. This lysis prevents bacteria from sheltering within the macrophages and promotes recruitment of neutrophils and other innate effectors to kill the bacteria. Thus, innate immune cells detect and control pathogens through multiple inflammatory approaches.

Along with inflammatory responses, immune cells must also survive long enough to respond to pathogens. All nucleated eukaryotic cells prevent lysis and plasma membrane disruption through membrane repair. Membrane repair is a poorly understood set of Ca^2+^ dependent processes that restore membrane integrity^45^. Following membrane disruption by a CDC like SLO, the cell activates at least two pathways, patch repair and intrinsic repair^16,45,46^. Patch repair is the hetero/homotypic fusion of internal vesicles with the plasma membrane, which patches the damaged site^46^. Intrinsic repair is the sequestration and shedding of toxins on microvesicles^16^. While these repair mechanisms help the cell by restoring membrane homeostasis, it is not clear if pathogens can exploit this repair process to promote immune evasion. Many immune activation receptors, including TLR4 and the IFNγ receptor (IFNγR), localize to cholesterol-rich microdomains^47–50^. PFO also localizes to cholesterol-rich microdomains^51^, so it is possible that intrinsic repair could remove immune receptors along with CDCs during repair. Several proteins are shed following CDC challenge, including the IL-6 receptor, and GPI-anchored proteins like CD14, alkaline phosphatase, and murine cytomegalovirus protein m157^16,52–54^. The functional consequences of shedding during intrinsic repair are unclear. It is possible that pathogens hijack membrane repair to block immune cell activation.

Here we tested the hypothesis that bacterial CDCs hijack membrane repair to suppress immune cell function. We found that the CDCs SLO and PFO temporarily impair macrophage responses to LPS and pro-inflammatory cytokines like IFNγ, as measured by TNFα production and surface expression of activation markers CD69 and CD86 without causing significant cell death. We found that TLR4 and IFNγR1 were both shed on microvesicles during intrinsic repair. In contrast, patch repair did not correlate with TNFα inhibition. Mutant toxins that enhanced membrane repair more potently inhibited macrophage responses. Overall, these findings suggest one mechanism for the immune evasion caused by *C. perfringens* and *S. pyogenes* during NSTI.

## Results

### CDCs functionally impair macrophages

**Figure 1:**
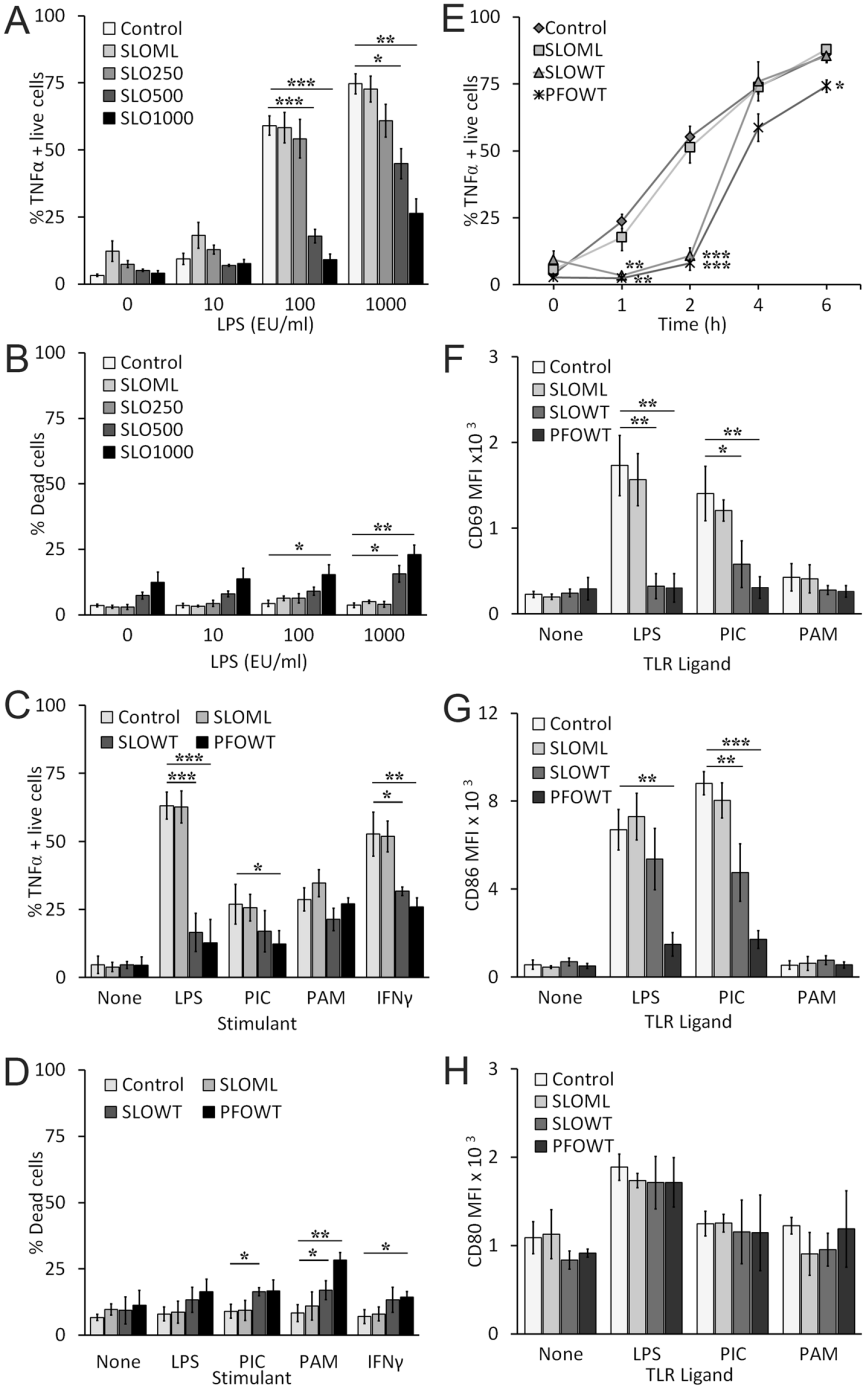
SLO impairs macrophage function. (**A**,**B**) BMDM were unchallenged (control) or challenged with 250 HU/mL, 500 HU/mL, 1000 HU/mL SLO WT or a mass of monomer-locked SLO (SLO ML) equivalent to the highest SLO WT dose for 10 min at 37 °C. Toxin was neutralized with serum. Cells were primed with the indicated concentrations of LPS for 2 h. BFA was added 30 min after LPS challenge. Cells were harvested, stained with Ghost Red 780, fixed, permeabilized, stained for intracellular TNFα and analyzed by flow cytometry. The percentage of TNFα positive cells gated on live cells (**A**) or the percentage of dead cells (**B**) is shown. (**C**,**D**) BMDM were unchallenged (control) or challenged with 500 HU/mL SLO WT, PFO or an equivalent mass amount of SLO ML for 10 min at 37 °C. Toxin was neutralized with serum. Cells were stimulated with no ligand, 100 EU/mL LPS, 10 µg/mL poly I:C (PIC), 1 µg/mL Pam3CSK4 (PAM), or 75 ng/mL IFNγ for 2 h. BFA was added 30 min after ligand challenge. Cells were harvested, stained and analyzed as in (**A**). The percentage of TNFα positive cells gated on live cells (**C**) or the percentage of dead cells (**D**) is shown. (**E**) BMDM were treated as in (**C**), except they were all stimulated with 100 EU/mL LPS for the indicated time points. BFA was added 30 min after LPS challenge. Cells were harvested, stained and analyzed as in (A). (**F–H**) BMDM were treated as in (**C**), except they were incubated with the indicated TLR ligands for 24 h and no BFA was added. Cells were harvested, stained with Ghost Red, then stained with anti-CD69 FITC (**F**), anti-CD86 APC (**G**), and anti-CD80 PE (**H**) and analyzed by flow cytometry. The mean fluorescence intensity (MFI) of living cells is shown. Graphs represent mean ± sem of at least three independent experiments. ***p < 0.001, **p < 0.01, *p < 0.05.

During a polymicrobial NSTI infection, both Gram positive and negative organisms could be present. To examine how CDCs could interact with other pathogen-associated molecular patterns that could be present during infection, we challenged murine C57BL/6 (B6) bone-marrow derived macrophages (BMDM) sequentially first with a CDC and then with a TLR ligand like LPS. We first determined the extent of TNFα production by BMDM to varying doses of LPS and the CDC SLO. BMDM were first challenged with three sublytic doses of SLO (250, 500, 1000 HU/mL), and then stimulated with varying doses of LPS for 2 h. We measured TNFα production by intracellular cytokine staining of live cells. We found that unstimulated and 10 EU/mL LPS did not induce TNFα production, but ~60% to 75% of BMDM produced TNFα when stimulated with 100 or 1000 EU/mL LPS (Fig. 1A). We found that 250 HU/mL SLO did not significantly alter TNFα production in BMDM treated with any dose of LPS, but 500 and 1000 HU/mL SLO caused a significant decrease in the proportion of TNFα positive live cells following 100 or 1000 EU/mL LPS stimulation (Fig. 1A). BMDM challenged with the pore deficient “monomer-locked” SLO (SLO ML) did not reduce TNFα production, indicating that this effect was not due to impurities in the toxin preparation and required oligomerization and/or pore formation of SLO (Fig. 1A). We next confirmed that SLO treatment was sublytic by measuring the proportion of dead cells in each population. Typically, cell death was <10%, except at the highest doses of LPS and SLO, which led to ~20% death (Fig. 1B). Based on this titration, we chose to perform future experiments with sublytic 500 HU/mL SLO followed by 100 EU/mL LPS exposure. Overall, these data suggest that SLO pretreatment may block TNFα production by BMDM.

We next determined the breadth of this functional impairment. We used a second CDC, PFO, because it is also involved in NSTI and immune evasion^4–8,23^. We further tested additional TLR ligands and cytokines that induce TNFα production in BMDM. We challenged BMDM with or without SLO ML, SLO WT or PFO for 10 min and then stimulated them without or with the TLR4 agonist LPS, TLR3 agonist polyinosinic-polycytidylic acid (poly I:C), TLR1/2 agonist Pam3SCK4, or IFNγ for 2 h and evaluated intracellular TNFα in live cells. We found that in response to LPS or IFNγ, PFO and SLO both similarly inhibited TNFα production (Fig. 1C). PFO, but not SLO, impaired TNFα production induced by TLR3 (Fig. 1C). Neither CDC blocked TNFα production induced by TLR2 (Fig. 1C). We measured necrosis in these cells and found that PFO and SLO were sublytic under most toxin/TLR ligand combinations. The greatest cytotoxicity was observed in BMDM treated with both CDCs and a TLR2 ligand (Fig. 1D). Importantly, TNFα production in these cells was not impaired, despite the increased cell death, indicating that cell death does not account for the attenuation of TNFα production. We next tested the time frame over which TNFα production was reduced. We challenged BMDM with CDCs, then exposed them to LPS for 0–6 h and measured TNFα production (Fig. 1E). We found that TNFα production was most potently inhibited at 2 h, but returned to regular levels 4 h after SLO challenge but was not fully restored following PFO challenge (Fig. 1E). Overall, these data show that CDCs from *S. pyogenes* and *C. perfringens* selectively and transiently inhibit TNFα production induced by LPS or IFNγ.

We next tested the extent of impairment in macrophages by examining surface expression of other activation markers and costimulatory proteins, like CD69, CD86 and CD80, following TLR stimulation. We found that LPS and poly I:C, but not Pam3SCK4, increased CD69, CD86 and CD80 surface expression (Fig. 1F–H), consistent with the Trif-dependent activation of costimulatory proteins^37^. Pretreatment with either SLO WT or PFO, but not SLO ML, reduced CD69 surface expression (Fig. 1F). PFO, but not SLO WT, significantly decreased CD86 surface levels (Fig. 1G). Interestingly, CD80 levels were not affected by either CDC (Fig. 1H). This suggests that PFO specifically targets immune activation because CD80 is associated with immune tolerance^55,56^. Overall, CDCs induced dysfunction of multiple innate immune mechanisms.

### Functional impairment of macrophages is not due to cell death

**Figure 2:**
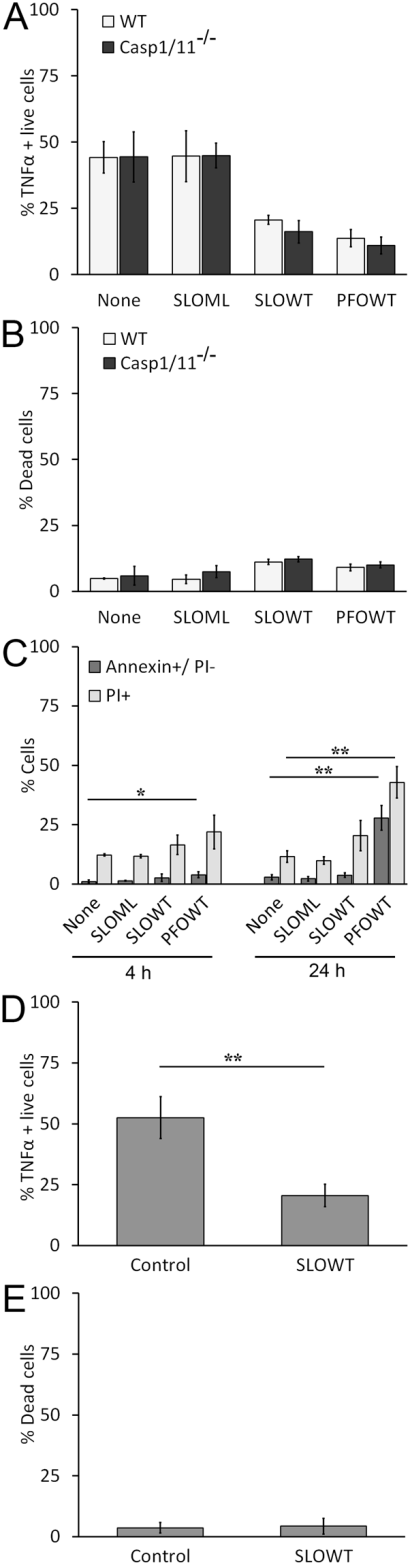
Functional impairment of macrophages by SLO is not due to cell death. (**A**,**B**) B6 (WT) or Casp1/11^−/−^ BMDM were treated as in Fig. 1C using 100 EU/mL LPS. The percentage of TNFα positive live cells (**A**) or percentage of dead cells (**B**) is shown. (**C**) BMDM were unchallenged or challenged with 500 HU/mL SLO WT, PFO or equivalent mass of SLO ML for 10 min, stimulated with 100 EU/mL LPS for 4 h or 24 h, harvested, stained with Annexin V and PI and analyzed by flow cytometry. The percentage of apoptotic (Annexin V^+^/PI^−^) or dead (PI^+^) cells is shown. (D-E) BMDM unchallenged or challenged with 500 HU/mL SLO for 10 min at 37 °C were sorted using a BD FACS Aria III to collect living cells. Sorted cells were stimulated with LPS and stained for TNFα as described in Fig. 1C. The percentage of TNFα positive cells gated on live cells (**D**) or the percentage of dead cells in this sorted population (**E**) is shown. Graphs represent mean ± sem of three independent experiments. **p < 0.01, *p < 0.05.

Although we limited our examination to cells with intact plasma membranes (eg Ghost Red impermeant), it is possible that the functional impairment we observed could be due to an alternative form of cell death or due to paracrine effects of Danger-Associated Molecular Patterns (DAMPs) released by the small subset of dying cells. We first examined alternative forms of cell death. We tested whether pyroptosis played any role in macrophage dysfunction by comparing the ability of CDCs to impair unprimed WT BMDM or pyroptosis-deficient Casp1/11^−/−^ BMDM. We found no difference in the ability of SLO WT or PFO to block TNFα production between WT BMDM and Casp1/11^−/−^ BMDM (Fig. 2A). The limited cell death we observed following CDC treatment was not due to pyroptosis, since both populations had a similar, low (<15%) extent of cell death (Fig. 2B). We next tested apoptosis, because other groups reported that SLO can induce apoptosis^22,24^. We measured the percentage of apoptotic cells by staining cells with Annexin V and propidium iodide (PI) after challenge with nothing, SLO ML, SLO WT, or PFO followed by LPS stimulation for 4 h or 24 h. PFO, but not SLO, caused a minor, but statistically significant, increase in apoptosis at 4 h following LPS stimulation (Fig. 2C). After 24 h, about 40% of PFO challenged cells were Annexin V^+^/PI^−^, whereas SLO WT still did not show any significant apoptosis (Fig. 2C). At 24 h, cell death due to necrosis was also elevated in cells pretreated with SLO WT or PFO (Fig. 2C). These data indicate that SLO does not impair macrophages through pyroptosis or apoptosis.

We next tested the possibility that macrophage impairment is mediated by DAMPs released by the small population of dead cells present following toxin challenge. To rule out interference from dead cells, we sorted living cells after SLO WT challenge but prior to LPS stimulation. We found that SLO WT impaired TNFα production even in the absence of dead cells (Fig. 2D). We confirmed that less than 5% of cell death occurred in the sorted cell population after 2 h of LPS stimulation (Fig. 2E). Overall, these data indicate that CDCs impair macrophages independently of cytotoxicity.

### Macrophages are impaired prior to TNFα mRNA synthesis

**Figure 3:**
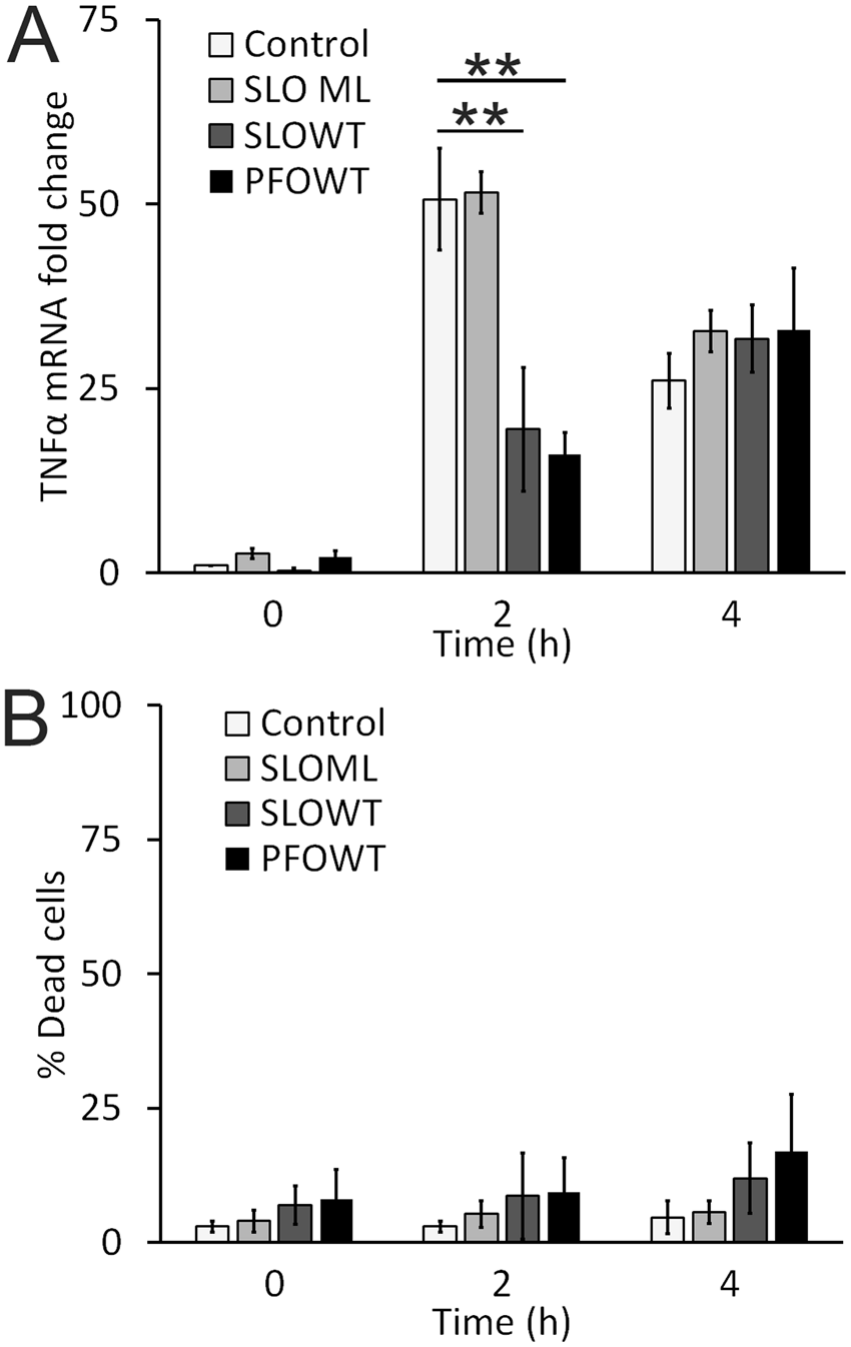
SLO prevents TNFα mRNA production. (**A**) BMDM were treated as in Fig. 1E, except they were all stimulated with 100 EU/mL LPS for 0, 2 or 4 h. Cells were harvested in Tri-Reagent and mRNA was isolated. cDNA prepared from individual mRNA samples was assessed for TNFα using real-time PCR. β-actin was used as an internal control. The fold change in relative TNFα mRNA expression level is shown. (**B**) A subset of BMDM from (**A**) were stained with PI instead of lysed in Tri-Reagent. Cell death was measured by flow cytometry. Graphs represent mean ± sem of three independent experiments. **p < 0.01.

Since macrophage dysfunction was not a consequence of cytotoxicity, we next determined the stage at which TNFα production is attenuated by CDCs. CDCs are pore-forming toxins, so it is possible that either TNFα protein or mRNA leaks out of the pores. Leakage of mRNA or protein from the cell can be tested by measuring both relative TNFα production and total RNA yield. If we measure a decrease in total RNA yield, it would indicate that mRNA leaked from the cell. If we measure no change in RNA yields or relative mRNA levels, it would indicate that TNFα production is blocked downstream of mRNA production. Finally, if we measure a specific decrease in TNFα mRNA, it would indicate that impairment occurs prior to TNFα mRNA synthesis and is not a result of mRNA or TNFα protein leakage from the cell. We challenged BMDM with nothing, SLO ML, SLO WT or PFO, then stimulated them with LPS for 2 h or 4 h and then measured their TNFα and β-actin mRNA expression by real-time PCR. We had normal RNA yields for all conditions. We observed a 50-fold increase in TNFα mRNA following 2 h of LPS stimulation and challenge with no toxin or SLO ML (Fig. 3A). In contrast, TNFα mRNA did not show a comparable relative increase in BMDM challenged with either SLO WT or PFO (Fig. 3A). Consistent with the temporary nature of the impairment, after 4 h of LPS stimulation, we observed no difference in relative mRNA levels between cells challenged and unchallenged with CDCs (Fig. 3A). To rule out the possibility that cell death accounts for the changes we observed in TNFα mRNA, we simultaneously measured cell death by PI staining a subsample of each group of cells. We found no significant amount of cell death due to CDC challenge and LPS stimulation under these conditions (Fig. 3B). These data suggest that the observed impairment is not due to leakage of TNFα mRNA or protein from the cell, but instead occurs prior to TNFα mRNA synthesis.

### Patch repair does not account for macrophage dysfunction

**Figure 4:**
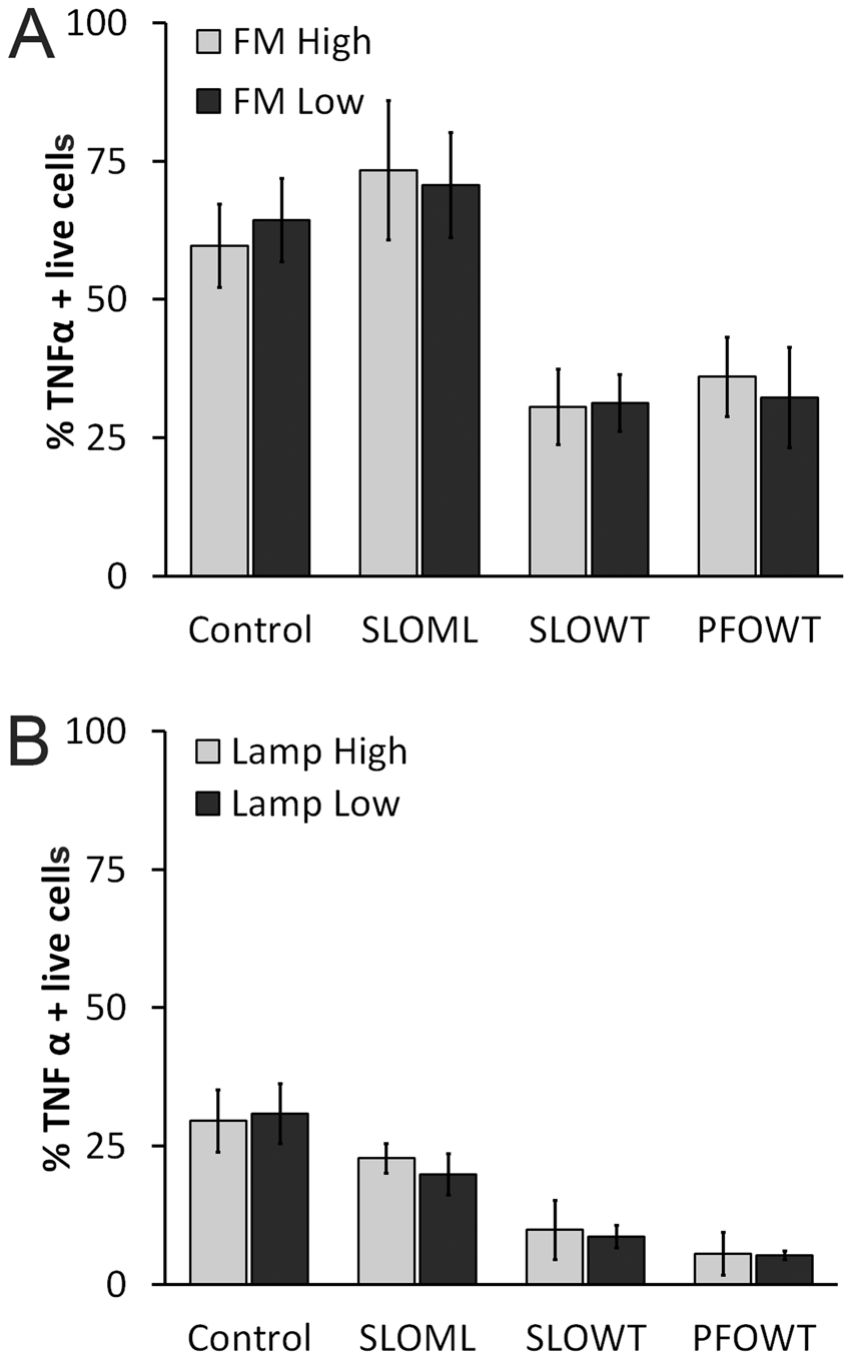
Patch repair and membrane permeabilization are not sufficient to impair macrophages (**A**) BMDM were unchallenged or challenged with 500 HU/mL SLO WT, PFO or equivalent mass amount of SLO ML for 10 min at 37 °C in presence of FM1–43FX. Excess toxin and dye were washed away with PBS. Cells were then primed with 100 EU/mL LPS for 2 h and stained for intracellular TNFα as described in Fig. 1A. The percentage TNFα positive live cells in FM1–43X^low^ and FM1–43X^high^ populations was measured by flow cytometry. (**B**) BMDM were challenged and stimulated as described in Fig. 2A. Cells were harvested, labeled with Ghost Red, stained for surface Lamp1, fixed, permeabilized and stained for intracellular TNFα. The percentage of TNFα positive live cells in Lamp1^low^ and Lamp1^high^ populations is shown. Graphs represent mean ± sem of three independent experiments.

Since impairment occurs prior to mRNA synthesis and is not due to TNFα leakage through toxin pores, it is possible that the functional impairment of macrophages is caused by processes that seal off and remove toxin pores—membrane repair. Two membrane repair mechanisms are used by cells to reseal lesions from CDCs: intrinsic repair and patch repair^16^. Intrinsic repair is the lipid-mediated sequestration and shedding of CDCs^16^, while patch repair is the Ca^2+^-dependent hetero- and homotypic fusion of internal vesicles and proteins that seal off the damage^45,46^. We first tested if patch repair drives impairment. We measured patch repair using two methods: entry of the membrane impermeant, fixable dye FM1–43X into the cell^57^ and surface staining of Lamp1 (CD107) to measure lysosomal fusion with the plasma membrane^58^. FM1–43X fluoresces only when bound to lipids. Increased FM1–43X fluorescence reflects an increased labeling of internal membranes. Brighter cells are those that took longer to reseal, demonstrating reduced patch repair capacity. If the extent of patch repair correlates with impairment, we predict FM1–43X^low^ cells would show a greater degree of TNFα inhibition. We challenged BMDM with nothing, SLO ML, SLO WT, or PFO for 10 min at 37 °C in presence of FM1–43X, washed, stimulated them with 2 h LPS and measured the proportion of TNFα positive live cells in FM1–43X^high^ and FM1–43X^low^ subsets. We found no difference in the proportion of TNFα positive live cells when we gated on either the FM1–43X^high^ or FM1–43X^low^ populations under any condition (Fig. 4A). This suggests that impairment does not correlate with patch repair in BMDM.

To confirm these results, we used a second approach to assess patch repair. During patch repair, internal membranes, including lysosomes, fuse with the plasma membrane^58,59^. As with the fusion of Natural Killer cell secretory granules^60,61^, this process elevates the levels of Lamp1 on the cell surface^58^. Cells undergoing extensive patch repair should show higher surface Lamp1 levels than other cells in the population. We measured surface Lamp1 expression on cells following CDC challenge and LPS stimulation. When we gated on Lamp1^high^ and Lamp1^low^ populations, we found SLO WT and PFO equally reduced TNFα production in both populations (Fig. 4B). Together, these data indicate that macrophage functional impairment does not correlate with patch repair.

### Activation receptors are shed following CDC challenge

**Figure 5:**
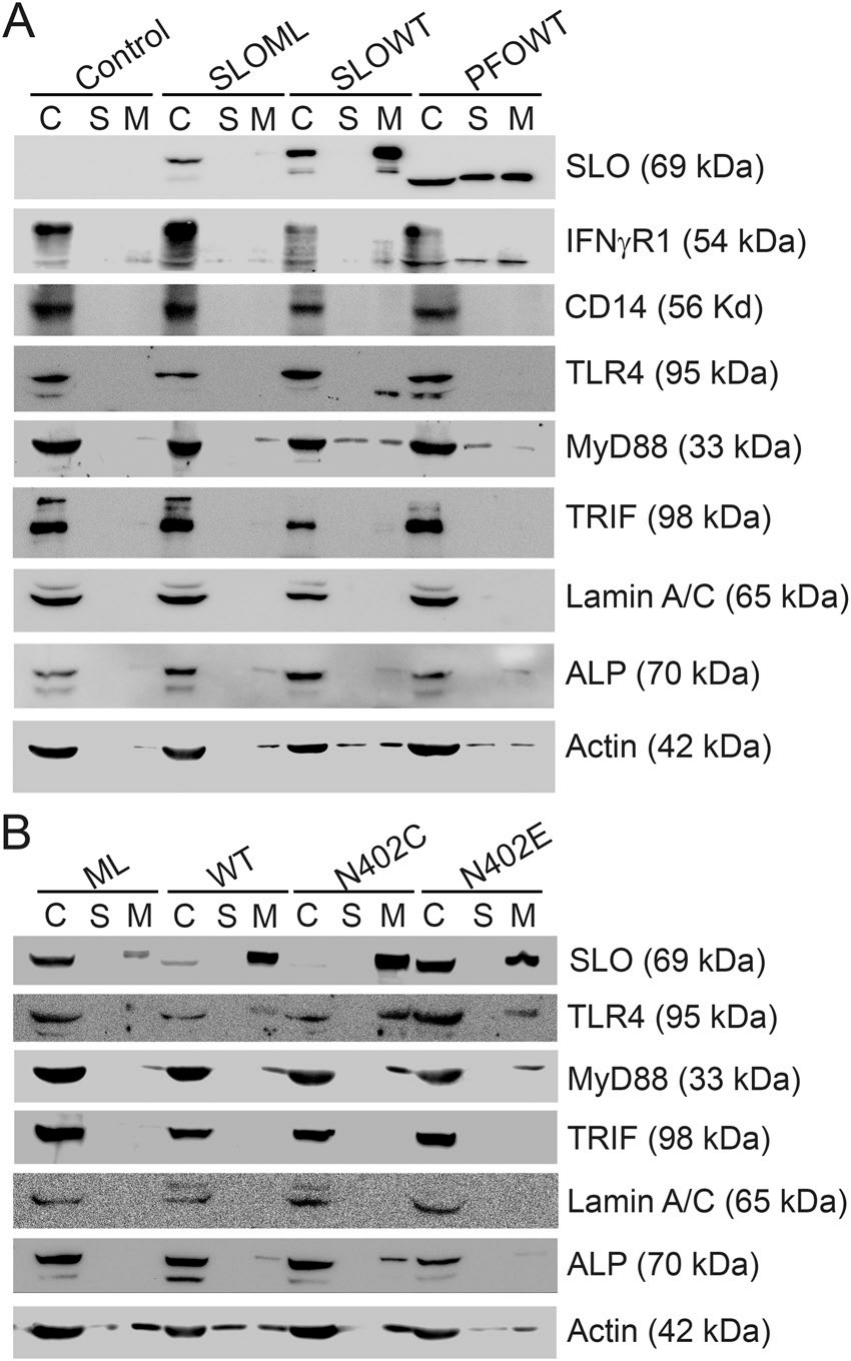
Activation receptors are shed during intrinsic repair. (**A**) BMDM were unchallenged (control) or challenged with 500 HU/mL SLO WT, PFO or an equivalent mass of SLO ML for 10 min at 37 °C and centrifuged at 2000 × g for 5 min to yield cell pellet (C). Supernatants were spun at 100,000xg for 40 min at 4 °C to collect the high speed supernatant (S) and microvesicle pellet (M). Samples were solubilized at 95 °C in SDS-sample buffer, resolved by SDS-PAGE and transferred to nitrocellulose. Portions of the blot were probed with 6D11 anti-SLO, anti-IFNγR1, 4B4F12 anti-CD14, 76B357.1 anti-TLR4, O91B8 anti-MyD88, 1H4B01 anti-Trif, EPR4477 anti-Alkaline Phosphatase, MANLAC-4A7 anti-Lamin A/C, and AC-15 anti-β-Actin antibodies followed by relevant secondary antibodies and ECL. Full-length blots are presented in Supplementary Figure S1. (**B**) BMDM were challenged with 500 HU/mL SLO WT or SLO N402C, SLO ML at equivalent mass to SLO WT, or SLO N402E at equivalent mass to SLO N402C for 10 min. Cell pellets (C), high speed supernatants (S) and microvesicles (M) were isolated as in (**A**) and probed with 6D11 anti-Streptolysin O, 76B357.1 anti-TLR4, O91B8 anti-MyD88, 1H4B01 anti-Trif, EPR4477 anti-Alkaline Phosphatase, MANLAC-4A7 anti-Lamin A/C, and AC-15 anti-β-Actin antibodies. Full-length blots are presented in Supplementary Figure S2. The blots are representative of at least 3 independent experiments.

Since patch repair did not correlate with macrophage impairment, we next tested if macrophage impairment is caused by intrinsic repair. Intrinsic repair is the sequestration and shedding of toxin molecules from the plasma membrane, so activation receptors could also be shed from the membrane during intrinsic repair. To test the hypothesis that key activation receptors are shed from BMDM along with active CDCs during intrinsic repair, we challenged BMDM with nothing, SLO ML, SLO WT, or PFO and then we isolated microvesicles (MV) from the cells. Consistent with previous results^16,52,54,62^, we found that SLO WT and PFO are shed on MV, whereas SLO ML, which does not trigger intrinsic repair, did not localize to MV (Fig. 5A, Supplementary Fig. S1). We measured activation receptors present in the MV fraction, and found that IFNγR1 was shed following SLO or PFO challenge, while TLR4 was shed following SLO challenge (Fig. 5A, Supplementary Fig. S1). In contrast to previous results^53^, we did not observe shedding of CD14 (Fig. 5A, Supplementary Fig. S1). We next examined the TLR4 signaling adaptors MyD88 and Trif. We found that SLO WT and PFO induced shedding of MyD88, but not Trif (Fig. 5A, Supplementary Fig. S1). To control for contamination of MV by cell debris, we verified that the plasma membrane associated, GPI-anchored protein Alkaline Phosphatase was shed in MV, while the nuclear envelope proteins Lamin A/C were not shed (Fig. 5A, Supplementary Fig. S1). We further determined the cholesterol to phospholipid ratio of MV. The high speed MV pellet from control cells had a cholesterol to phospholipid ratio of 0.092 ± 0.047 (mean ± sem), while the ratio for MV shed from SLO- or PFO-challenged cells was 0.296 ± 0.049 and 0.363 ± 0.040, respectively (p = 0.0398 and 0.012 vs control by ANOVA). Values for MV from SLO- or PFO-challenged BMDM are consistent with previous measurements of the cholesterol to phosopholipid ratio for the plasma membrane^63,64^. Also consistent with previous results^16^, β-Actin was present in all three fractions following treatment with cytotoxic CDCs (Fig. 5A, Supplementary Fig. S1). These data suggest that CDCs trigger shedding of activation receptors during intrinsic repair.

**Figure 6:**
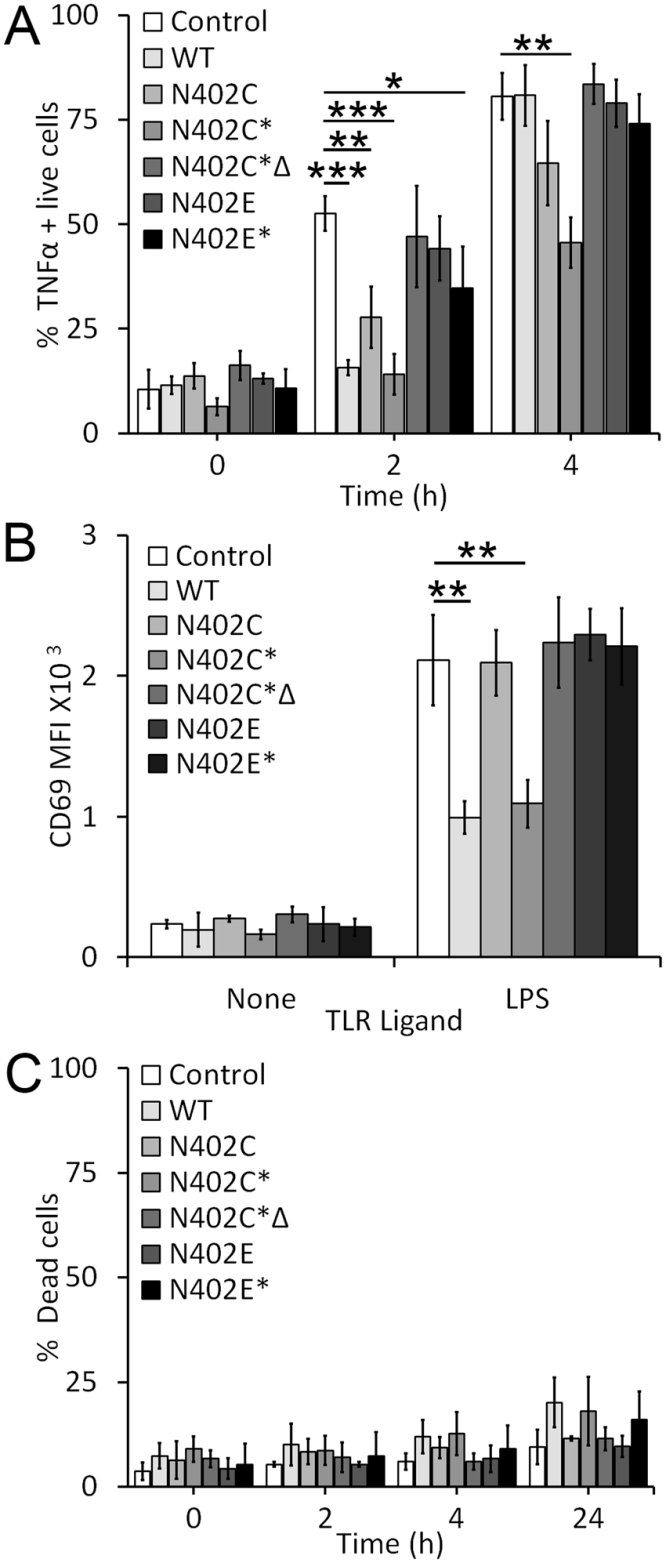
Pore formation enhances SLO mediated functional impairment of macrophages. (**A**) BMDM were unchallenged or challenged for 10 min at 37 °C with 500 HU/mL SLO WT, an equivalent mass amount of SLO N402C, SLO N402E, a 10-fold mass excess of either SLO N402C (equivalent hemolytic dose) (N402C*), heat inactivated SLO N402C (N402C*∆) or SLO N402E (N402E*), primed with LPS for the indicated times in presence of BFA, stained with Ghost Red and then stained for intracellular anti-TNFα as described in Fig. 1A. The percentage of TNFα positive cells gated on live cells is shown. (**B**) Cells challenged as in (**A**) were stimulated with LPS overnight without BFA and stained with Ghost Red followed by anti-CD69 FITC as described in Fig. 1F. The mean fluorescence intensity of CD69 from live cells is shown. (**C**) The percentage of dead cells (Ghost Red positive) at 0, 2, 4 or 24 h is shown. Graphs represent mean ± sem of at least three independent experiments. ***p < 0.001, **p < 0.01, *p < 0.05.

We next tested if we could change the extent of activation receptor shedding by controlling the extent of intrinsic repair. In order to control the extent of intrinsic repair, we used the well-characterized SLO mutants SLO N402E (array-locked SLO) and N402C (SLO N402C)^15,16,18^. Array-locked SLO triggers reduced intrinsic repair compared to SLO WT, while the mixture of pores and linear oligomers formed by SLO N402C triggers increased intrinsic repair at equivalent hemolytic doses^15,16^. We challenged BMDM with SLO ML, SLO WT, an equivalent hemolytic dose of SLO N402C, or an equivalent mass of array-locked SLO to SLO N402C and isolated MV. As expected, we found that SLO N402C was more extensively shed than SLO WT, while array-locked SLO was not as extensively shed, and SLO ML was minimally shed (Fig. 5B, Supplementary Fig. S2). We measured the shedding of the TLR4 pathway under these conditions, and found that TLR4 and MyD88 were more extensively shed following SLO N402C challenge, and less so with array-locked SLO (Fig. 5B, Supplementary Fig. S2). Trif was not shed under any condition (Fig. 5B, Supplementary Fig. S2). Alkaline Phosphatase shedding was similar to TLR4 and MyD88, confirming that SLO N402C induced more shedding, while array-locked SLO induced less shedding than SLO WT (Fig. 6B). Lamin A/C was found strictly in the cell lysate, indicating cellular integrity in all samples (Fig. 5B, Supplementary Fig. S2). Overall, these findings suggest that TLR4 and MyD88 are shed by intrinsic repair.

### Functional impairment requires pore formation

We next tested the relative contribution of receptor shedding to macrophage impairment. We compared the extent of TNFα production following SLO challenge with our various toxin mutants and LPS stimulation. Since SLO N402C is 10-fold less hemolytic than SLO WT^15^, we used both an equivalent mass of SLO N402C or an equivalent hemolytic dose of SLO N402C (N402C*) to SLO WT. We found that both SLO WT and an equivalent mass of SLO N402C reduced the proportion of TNFα positive cells at 2 h, but not at 4 h (Fig. 6A). However, an equivalent hemolytic dose of SLO N402C impaired TNFα production at both 2 and 4 h (Fig. 6A). Heat inactivation of SLO N402C completely reversed this effect, indicating that functional SLO N402C was needed (Fig. 6A). However, intrinsic repair alone may not be sufficient for this phenotype, since an equivalent mass of array-locked SLO to SLO N402C did not impair TNFα production to the same degree in LPS stimulated BMDM (Fig. 6A). We next tested the ability of these mutant toxins to block CD69 expression (Fig. 6B). Similar to SLO WT, SLO N402C* reduced CD69 expression in BMDM (Fig. 6B). In contrast, the equivalent mass amount of SLO N402C, heat inactivated SLO N402C* or array-locked SLO at any dose failed to block CD69 induction (Fig. 6B). To control for cell death, we measured cytotoxicity in our samples. We found that all mutant toxins killed an equal or reduced number of cells compared to SLO WT (Fig. 6C). These data suggest that pore formation is necessary for the macrophage impairment.

## Discussion

In this study, we describe the ability of CDCs to suppress immune cells by blocking pro-inflammatory macrophage responses. This blockade appears to be cell intrinsic and mediated by the membrane repair response to CDCs, specifically by the shedding of activation receptors during intrinsic repair. We propose a model of host-pathogen interactions starting with an attempt by pathogenic bacteria to kill cells using pore-forming toxins. Cells resist toxin lethality via membrane repair. Bacteria then hijack membrane repair to force elimination of surface immune activation receptors like TLR4 and IFNγR1. Immune cells respond with intracellular activation receptors like NLRP3 to secrete DAMPs and cytokines like IL-1β. Bacteria in turn encode separate proteins to block DAMPs and cytokines. Together, these results provide mechanistic insight into CDC-mediated immune evasion mechanisms. These immune evasion mechanisms may contribute to the pathogenicity and mortality of NSTI.

We found that CDCs promote the shedding of activation receptors on microvesicles. This extends the previous body of work showing that the IL-6 receptor and GPI-anchored proteins are shed by SLO^16,52–54^. Although a previous study observed the shedding of the GPI-anchored CD14^53^, to our surprise, we did not observe CD14 shedding. It is possible that this difference is due to assay conditions or to the use of murine vs human macrophages. Regardless, our results link immune receptor shedding to intrinsic membrane repair. We found that intrinsic repair was more important for the immune suppression phenotype than patch repair. This is consistent with our mechanism of receptor elimination by the shedding that occurs during intrinsic repair, and not by the hetero/homotypic fusion of vesicles that occurs during patch repair. However, our finding that pore-competence was necessary for the functional impairment of macrophages suggests that patch repair or other internal events may still enhance immune suppression. Future work can address the mechanism by which pore competence induces immune suppression.

We conclude from our data that immune suppression required pore formation. However, we cannot rule out the possibility that there is a threshold requirement for activation receptors. It is possible that array-locked SLO, which also promotes intrinsic repair^16^, does not promote sufficient shedding to trigger the same degree of immune suppression. However, we think this is unlikely based on our western blots showing a similar extent of TLR4 shedding. Interestingly, we never observed complete removal of either TLR4 or IFNγR1. It is possible that the remaining cell-associated receptors are not capable of signaling, or that a certain receptor density is required for productive signaling. It is also possible that in addition to receptor shedding, CDCs disrupt the ability of these receptors to signal. Both CDCs we examined, SLO and PFO, form 50–70 subunit pores^14^. Each subunit can theoretically engage cholesterol and CDCs localize to cholesterol-rich microdomains^51,65,66^. This has the potential to disrupt cholesterol-rich microdomain organization, especially when multiple toxin pores are sequestered in a relatively limited space. Disruption of cholesterol-rich microdomains can abrogate both TLR4 and IFNγ signaling^48–50^. It is possible that in addition to shedding, CDCs further disrupt signaling by altering cholesterol-rich microdomain organization. Interestingly, TLR4 localization to cholesterol-rich microdomains is enhanced by MyD88 dependent TLR4 signaling^67^. Under these circumstances, we predict this would increase the amount of TLR4 shed and/or present in the disrupted area. Indeed, a previous report suggests that membrane repair interferes with B cell receptor signaling^68^, though they did not examine the contribution of B cell receptor shedding to their phenotype. Future work will aim to dissect the contribution of cholesterol-rich microdomain remodeling and microvesicle shedding to the regulation of cell signaling and membrane repair.

Membrane repair is fundamentally a cell protective response. During CDC attack, membrane repair is critical for maintaining cell viability^54,62^. In some cases, membrane repair further promotes immune responses^15,69^. For example, we have previously shown that mutant toxins which provoke increased repair responses and reduced lethality (e.g. SLO N402C) lead to increased IL-1β secretion^15^. In this circumstance, membrane repair preserves the cell long enough for it to execute a pro-inflammatory pyroptotic program^15^. Similarly, during necroptosis, membrane repair prolongs cell survival from the pore-forming toxin Mixed Lineage Kinase-Like long enough to promote chemokine secretion and cross-presentation to T cells^69^. Overall, membrane repair is generally beneficial to the host.

However, membrane repair can also be subverted by pathogens to attenuate the immune response. Previously, immune evasion has been attributed to CDCs, including both SLO^21,22,29^ and PFO^4–8,23^. Both CDCs are critical for pathogen virulence *in vivo*^5,9,23^. Although other groups have suggested that necrosis or apoptosis^9,21,22^ may underlie immune evasion, we show here that macrophages, like neutrophils^29^, can be inactivated without their death. We suggest that the mechanism of immune suppression occurs at least partially through the shedding of activation receptors on microvesicles. We show that TLR4 and IFNγR1 are shed, while previous work has suggested IL-6 receptor and CD14 are shed^53^. Although TNFα production is only transiently (2–4 h) impaired, we observed long-term effects on CD69 and CD86 surface expression. The selective blockade of CD86 instead of CD80 suggests that pathogens like *C. perfringens* could potentially further promote immunosuppression by altering the relative surface expression of CD86 and CD80 on antigen-presenting cells. Taken all together, these immune evasion strategies could account for the accumulation of immune cells that do not enter necrotic tissue during gas gangrene^4–8^. In contrast, during necrotizing fasciitis, macrophages infiltrate the infected area, but are inactivated and thus unsuccessful in clearing the bacteria^30,31^. Overall, CDCs hijack membrane repair to promote immune evasion.

In response to these immune evasion strategies, we note that immune cells can sense and respond to CDCs with alternative pro-inflammatory mechanisms. The cytoplasmic sensor NLRP3 detects membrane disruption induced by CDCs, leading to Casp1 and inflammasome activation. As a result, CDC pore-formation potently induces IL-1β^15,40,41^. However, bacteria have evolved separate mechanisms to counter this pro-inflammatory response. In the case of *S. pyogenes*, SLO is closely associated with a second virulence factor, NAD^+^-glycohydrolase (Spn)^10–13^. Spn significantly enhances SLO-mediated cytotoxicity, but it also inactivates IL-1β^11^. Inactivation of IL-1β allows *S. pyogenes* to evade this immune response. This illustrates how membrane repair fits into the wider context of host-pathogen interactions.

These host-pathogen interactions may be more specific for rapid NSTIs. Previous studies claimed that CDCs from *S. pneumoniae* and *Bacillus anthracis* activate TLR4 and promote apoptosis^70–72^, which is the opposite of what we find here. One explanation for the discrepancy in results is that we used different toxins and different time points. We examined earlier time points likely to be more relevant during NSTI. Typically, other studies examined TNFα mRNA at least 4 h after pneumolysin (*S. pneumoniae*) or anthrolysin O (*Bacillus anthracis*) challenge, which is more relevant for the diseases caused by those pathogens^70–72^. If CDCs bind to TLR4, it is possible that they act as weaker agonists and/or block LPS-dependent TLR4 activation, requiring longer times to promote activation. Alternatively, immune cells might enhance TNFα production at later time points in an attempt compensate for earlier inhibition. Future studies could examine the temporal response of TNFα to different bacterial CDCs to determine the relative contributions of binding, timing and toxin structure to this discrepancy. Regardless, all of these studies serve to underscore the importance of TNFα production during pathogen control, and delineate steps both host and pathogen use to control TNFα production.

Overall, we propose that bacteria hijack the shedding that occurs during intrinsic membrane repair. This becomes an immune evasion mechanism by which TNFα production and the surface expression of activation markers and costimulatory receptors are blocked. This mechanism may account for the pathophysiology and mortality caused by *C. perfringens* and *S. pyogenes*. Although our system models cooperative polymicrobial immune evasion by using LPS (Gram negative pathogens) and a CDC (Gram positive pathogens) that occurs during some NSTIs, it is possible that these pathogens also target TNFα induction by proinflammatory cytokines during single microbe infections. Both *C. perfringens* and *S. pyogenes* are lethal single agents in NSTI. *S. pyogenes* also causes septic shock, and septic cardiomyopathy. This suggests that intrinsic membrane repair may serve as one key host-pathogen interface in a broad range of diseases.

## Materials and Methods

### Reagents

All reagents were from Thermofisher Scientific (Waltham, MA) unless otherwise noted. The pBAD-gIII plasmid encoding His-tagged SLO was a kind gift from Michael Caparon (Washington University in St Louis, St Louis, MO)^17^. Cysteine-less His-tagged PFO in pET22 was a generous gift from Rodney Tweten (University of Oklahoma Health Sciences Center, Oklahoma City, OK)^73^. Cysteine-less (C530A) SLO, monomer-locked (G398V/G399V) SLO, array-locked (N402E) and N402C SLO were previously described^15,16^. Anti-Lamin A/C was obtained from the Developmental Studies Hybridoma Bank, created by the NICHD of the NIH and maintained at The University of Iowa, Department of Biology, Iowa City, IA 52242. Anti-Lamin A/C (MANLAC-4A7-s) was deposited to the DSHB by G.E. Morris (DSHB Hybridoma Product MANLAC1(4A7)). Anti-Alkaline Phosphatase EPR4477 rabbit monoclonal antibody (rAb) (Catalog: GTX62596) and anti-β-actin AC-15 mouse monoclonal antibody (mAb) (Catalog: GTX26276) were obtained from GeneTex (Irvine, CA). Anti-IFNγR1 goat polyclonal antibody (Catalog: 1026-GR) was from R&D Systems (Minneapolis, MN). Anti-Streptolysin O 6D11 mAb (Catalog: NBP1–05126), anti-CD14 4B4F12 mAb (Catalog: NBP2–37291), and anti-TLR4 76B357.1 rAb (NB100–56566SS) were from Novus Biologicals (Littleton, CO). Anti-MyD88 O91B8 mAb (Catalog: 675802), anti-Trif 1H4B01 mAb (Catalog: 657102), FITC conjugated anti-CD69 H1.2F3 (Catalog: 104506), PE-conjugated anti-CD80 16–10A1 (Catalog: 104708), APC-conjugated anti-CD86 GL-1 (Catalog: 105012), FITC-conjugated anti-TNFα MP6-xT22 (Catalog: 506304), FITC-conjugated anti-Lamp1 1D4B (Catalog: 121608) antibodies and Brefeldin A were from BioLegend (San Diego, CA). HRP-conjugated anti-mouse (Catalog: 711–035–151), anti-rabbit (Catalog: 711–035–152) and anti-Goat (Catalog: 705–035–147) antibodies were from Jackson Immunoresearch (West Grove, PA). Ghost Red-780 was from Tonbo Biosciences (San Diego, CA). Ultrapure LPS, poly I:C and Pam3CSK4 were from Invivogen (San Diego, CA). IFNγ recombinant protein was from eBiosciences (San Diego, CA).

### Recombinant Toxins

Toxins were purified as previously described^16,74^. Briefly, log-phase *E. coli* BL21 were induced with 0.2% arabinose (SLO), or 0.2 mM IPTG (PFO) for 3 h at room temperature and purified using Nickel-NTA beads. Protein concentration was determined by Bradford Assay and hemolytic activity was determined as previously described^16,74^. Specific activity for toxins was 4.5 × 10^5^–1.28 × 10^6^ HU/mg for SLO WT, 8.82 × 10^6^ HU/mg for PFO, 4.8 × 10^4^ HU/mg for SLO N402C. Pore-deficient toxins SLO G398V/G399V (SLO monomer-locked), and SLO N402E (SLO array-locked) had a specific activity of <10 HU/mg. The sublytic dose of toxin used was the highest concentration of toxin that killed <20% of target cells. For BMDM, this was 500 HU/mL, consistent with our previous results^15,16^.

### Mice

All mice were housed and maintained at Texas Tech University, adhering to the Guide for the Care and Use of Laboratory Animals (8th Ed., NRC 2011) for animal husbandry. All experimental procedures were approved by the Texas Tech University IACUC. Casp1/11^−/−^ mice on the C57BL/6 background (stock: 016621) and wild type C57BL/6 mice (stock: 000664) were purchased from The Jackson Laboratory (Bar Harbor, ME). Mice of both genders aged 6–15 weeks were used to prepare BMDM. Sample size was determined as the minimum number of mice needed to provide sufficient bone marrow for experiments. Consequently, no randomization or impairment was needed. Euthanasia was performed by asphyxiation through controlled flow of pure CO_2_ followed by cervical dislocation.

### Cell Culture

Bone-marrow derived macrophages (BMDM) were isolated from femora and tibiae and cultured as previously described^75^. Briefly, BMDM were differentiated at 37 °C, 5% CO_2_ for 7–21 days in DMEM supplemented with 30% L929 cell supernatant, 20% FCS (VWR Seradigm, Radnor, PA), 1 mM sodium pyruvate (Corning, Corning, NY), 1x Penicillin/Streptomycin (Hyclone, Logan, UT), and 1x L-glutamine (Hyclone).

### TNFα induction and FACS staining of cells

BMDM were unchallenged (control) or challenged with a sublytic dose of SLO WT, PFO, SLO N402C, or a mass of SLO ML or SLO N402E equivalent to the highest SLO WT or SLO N402C dose for 10 min at 37 °C in RPMI media supplemented with 2 mM CaCl_2_. Toxin was neutralized by addition of 10% FCS, followed by dilution with RPMI and centrifugation at 500 × g for 10 min. Cells were then resuspended in RPMI supplemented with 10% FCS, 1x Penicillin/Streptomycin and 1x L-glutamine (R10) and stimulated with 100 endotoxin units (EU)/mL LPS, 10 µg/mL poly I:C, 1 µg/mL Pam3CSK4, or 75 ng/mL IFNγ for 2 h unless otherwise mentioned. Brefeldin A (BFA) was added at 5 μg/mL 30 min after stimulation. Cells were harvested in PBS, stained with 1 µL Ghost dye Red-780 for 30 min on ice and washed in FACS buffer. Cells were then fixed in 2% paraformaldehyde for 15 min on ice, permeabilized using 0.1% saponin in FACS buffer for 10 min on ice, washed and stained with 5 μg/mL anti-TNFα conjugated to FITC for 40 min on ice, washed and analyzed in FACS buffer on an Accuri C6 (BD Biosciences, San Jose, CA). The percentage of TNFα positive cells was determined after gating on live cells, while the percentage of dead cells was determined either by the Ghost Red high population or PI staining of a subsample. For other surface marker staining, cells were challenged with CDCs and stimulated with LPS or other agonists as described above. However, cells were stimulated without BFA, incubated overnight instead of 2 h, neither fixed nor permeabilized but instead surface stained for 30 min on ice with anti-CD69 FITC, anti-CD80 PE and anti-CD86 APC conjugated antibodies. The mean fluorescence intensity (MFI) of the respective surface marker was determined from live cells and percentage of dead cells was determined by the percentage Ghost Red^high^ cells using a FACS Aria III (BD Biosciences, San Jose, CA).

### Cell Sorting

BMDM were unchallenged or challenged with 500 HU/mL SLO WT for 10 min at 37 °C in RPMI supplemented with 2 mM CaCl_2_. Toxin was neutralized by addition of 10% FCS followed by dilution in RPMI. Cells were centrifuged at 500 × g for 10 min, resuspended in PBS containing 2% FCS and 20 µg/mL PI and the PI negative population was sorted on a FACS Aria III (BD Biosciences). Sorted cells were resuspended in R10 and stimulated with 100 EU/mL LPS for 2 h followed by intracellular cytokine staining with APC-conjugated anti-TNFα as described above.

### Apoptosis Assay

BMDM were unchallenged or challenged with SLO WT, PFO or a mass equivalent of SLO ML for 10 min at 37 °C followed by 2, 4 or 24 h of stimulation with 100 EU/mL LPS at 37 °C. Following stimulation, cells were stained with 0.9 µg/mL Annexin V-FITC and 20 µg/mL PI in 1.4 mM NaCl, 25 mM CaCl_2_, 0.1 M HEPES, pH 7.4 for 15 min on ice and analyzed by FACS using an Accuri C6.

### Membrane permeabilization and repair

BMDM were unchallenged or challenged with 500 HU/mL SLO WT, PFO or equivalent mass amount of SLO ML for 10 min at 37 °C in the presence of FM1–43X, then washed with PBS to remove excess toxin and dye, resuspended in R10, stimulated with 100 EU/mL LPS for 2 h and stained using APC anti-TNFα as described above. The percentage of TNFα positive cells were determined from both FM1–43X^low^ (high repair) and FM1–43X^high^ (low repair) populations. Alternatively, BMDM were challenged with CDCs in the absence of FM1–43FX for 10 min at 37 °C, resuspended and stimulated with 100 EU/mL LPS as described above. Cells were harvested, stained with Ghost Red, then FITC anti-LAMP1, followed by fixation, permeabilization and staining as described above for intracellular TNFα cytokine staining. The percentage of TNFα positive cells were determined from live cells with either low or high Lamp1 staining.

### Isolation of Microvesicles

Microvesicles were isolated as previously described^54^. Briefly, 5 × 10^6^–1 × 10^7^ cells were harvested, resuspended in RPMI media supplemented with 2 mM CaCl_2_ to 2.5 × 10^6^–5 × 10^6^ cells/mL, challenged with a sublytic concentration of toxin (hemolytic toxins) or equivalent mass (inactive toxins) and incubated for 15 min at 37 °C. Cells were pelleted at 2,000 × g for 5 min, solubilized at 95 °C in SDS-sample buffer for 5 min and sonicated. Supernatants were spun at 100,000 × g in a Beckman Coulter TL-100 ultracentrifuge using a Beckman TLS 55 rotor for 40 min at 4 °C. Both high-speed supernatant and microvesicle pellet were solubilized at 95 °C in 4 × SDS-sample buffer for 5 min.

### SDS-PAGE and Immunoblotting

The low speed cell pellet, high speed supernatant, and microvesicle samples from BMDM challenged with CDCs as described above were resolved on 10% polyacrylamide gels at 90 V for 165 min and transferred in an ice bath to nitrocellulose in transfer buffer (15.6 mM Tris and 120 mM glycine) at 90 mA for 85 min. Blots were blocked using 5% skim milk in 10 mM Tris-HCl, pH 7.5, 150 mM NaCl and 0.1% Tween 20 (TBST), incubated with 6D11 anti-SLO mAb (1:1000), 4B4F12 anti-CD14 mAb (1:1000), 76B357.1 anti-TLR4 rAb (1:500), O91B8 anti-MyD88 mAb (1:1000), MANLAC-4A7 anti-Lamin A/C mAb (1:250), AC-15 anti-β Actin mAb (1:5000), 1H4B01 anti-Trif rAb (1:1000), EPR4477 anti-Alkaline Phosphatase rAb (1:1000) or anti-IFNγR1 goat polyclonal antibody (1:1000) in 1% skim milk in TBST followed by HRP-conjugated anti-mouse, anti-rabbit or anti-goat IgG antibodies in 1% skim milk in TBST and developed with ECL (0.01% H_2_O_2_ (Walmart, Fayetteville, AR), 0.2 mM p-Coumaric acid (Sigma), 1.25 mM Luminol (Sigma), 0.1 M Tris pH 8.4).

### Lipid Assays

Microvesicles were isolated as described above from BMDM challenged with SLO or PFO. Lipids were extracted from the microvesicle pellets using 200 μL methanol for 30 min at room temperature and centrifuged at 12,000 × g for 2 min at room temperature. Supernatants were assayed for total cholesterol content (Catalog: 10007640, Cayman Chemicals, Ann Arbor, MI) and phospholipid content (Catalog: EPLP-100, BioAssay Systems, Hayward, CA) according to manufacturer’s instructions. Fluorescence was read using 535 excitation and 585 emission filters on a FL600 fluorescence microplate reader (Biotek, Winooski, VT).

### RT-PCR

Cell were treated with toxins as described above followed by 2 or 4 h of stimulation with 100 EU/mL LPS at 37 °C. Total RNA was extracted using TRI-Reagent. cDNA was generated using Superscript III. TNFα and β-actin levels were measured on an ABI 7300 using SybrGreen and compared using ΔΔCt as previously described^76^. Primer sequences for β-actin are 5′-GAAATCGTGCGTGACATCAAAG and 5′-TGTAGTTTCATGGATGCCACAG and for TNFα are 5′-CCTGTAGCCCACGTCGTAG and 5′-GGGAGTAGACAAGGTACAACCC.

### Statistics

Origin software (Northampton, MA) was used for statistical analysis. Data are represented as mean ± SEM. Statistical significance was determined by Two-way ANOVA with Bonferroni post-testing. p < 0.05 was considered statistically significant. Graphs were generated in Excel (Microsoft, Redmond, WA) and Photoshop (Adobe Systems Incorporated, San Jose, CA).

### Data availability

The datasets generated during and/or analyzed during the current study are available from the corresponding author on reasonable request. Full western blots used to construct Fig. 5 are available as Supplementary Data (Supplementary Figs S1–S2).

## Electronic supplementary material

Supplemental Figures S1 and S2
