# Supplementary material for: Cholesterol-dependent cytolysins impair pro-inflammatory macrophage responses
Source: Sci Rep. 2018 Apr 24;8:6458. doi: 10.1038/s41598-018-24955-2 (PMC5915385; doi:10.1038/s41598-018-24955-2)
Supplement: Supplementary file 1 — Supplemental Figures S1 and S2 [file 41598_2018_24955_MOESM1_ESM.pdf]

Cholesterol-dependent cytolysins impair pro-inflammatory macrophage responses

Pushpak Bhattacharjee[1,†] and Peter A Keyel[1, *]

[1]Department of Biological Sciences
Texas Tech University
Lubbock, TX 79409

[†]Current address:
Department of Cell Biology and Biochemistry
Texas Tech University Health Science Center
Lubbock, TX 79430

[*]to whom correspondence should be addressed:
Peter Keyel
Department of Biological Sciences
Biology Rm 108
Box 43131
Lubbock, TX 79409-3131
tel: (806) 834-6248
fax: (806) 742-2963
email: peter.keyel@ttu.edu

Supplemental Material

Supplemental Figure S1
Supplemental Figure S2

Supplemental Figure S1

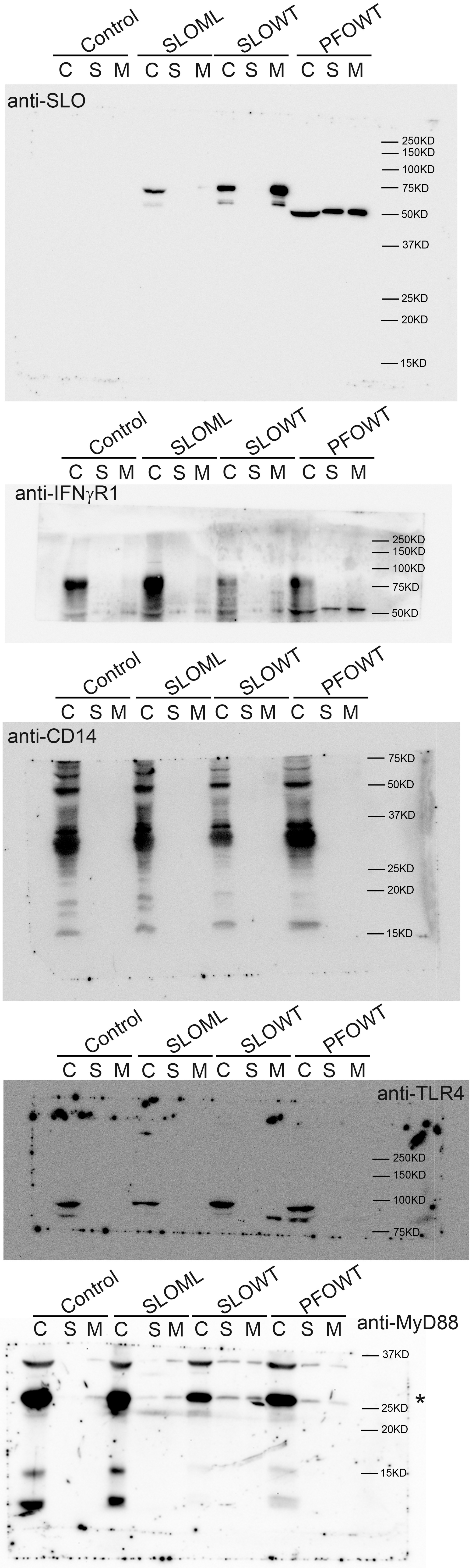

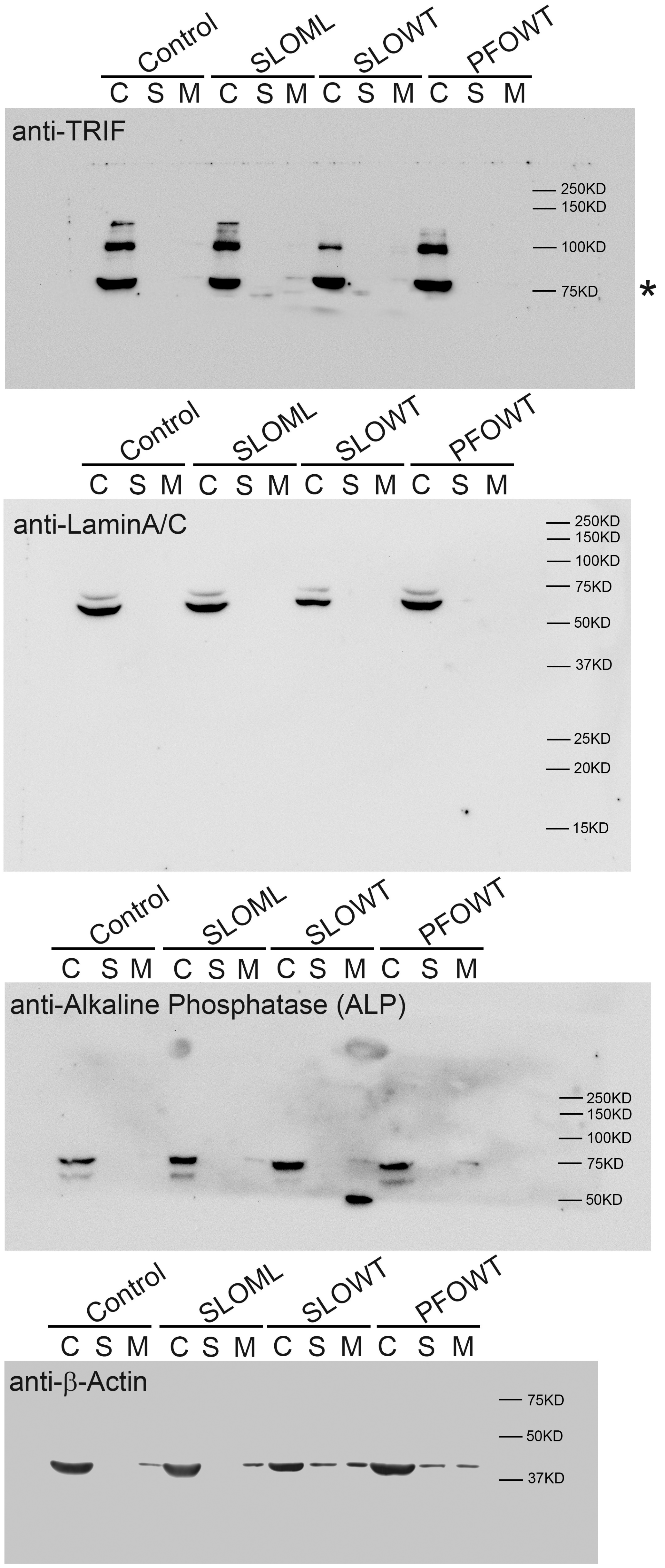

Supplementary Figure S1. Activation receptors are shed during intrinsic repair. The full western blots from Fig 5A are shown. In many cases, the blots were cut following Ponceau S stain and prior to staining with primary antibody. The * denotes bands remaining due to incomplete stripping of prior antibodies. C = cell pellet, S = high speed supernatant, M = microvesicle pellet. Size in kilodaltons (KD) is shown.

Supplemental Figure S2

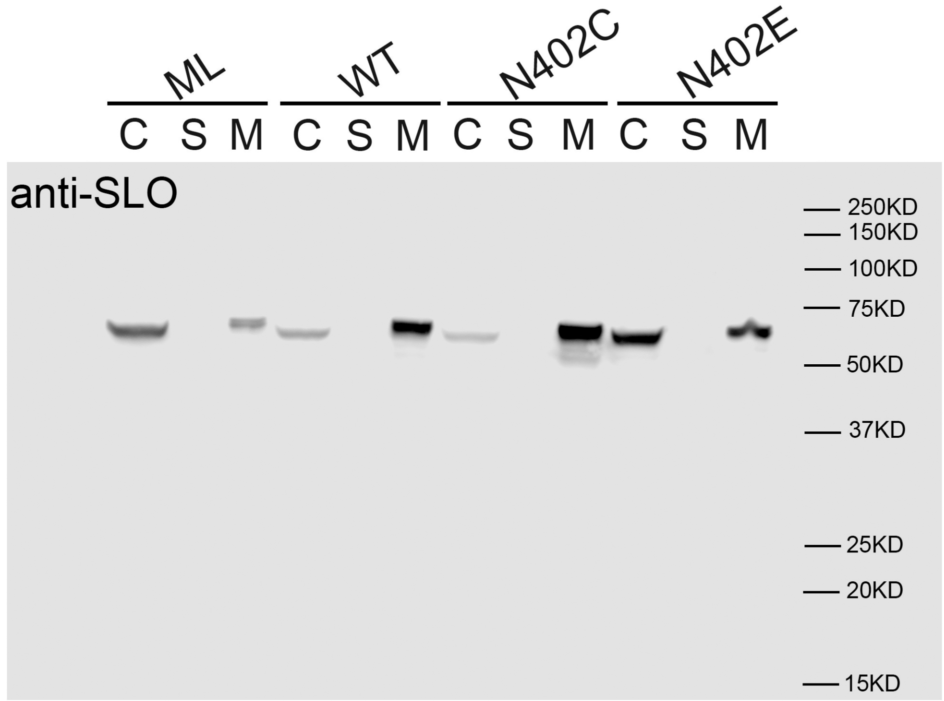

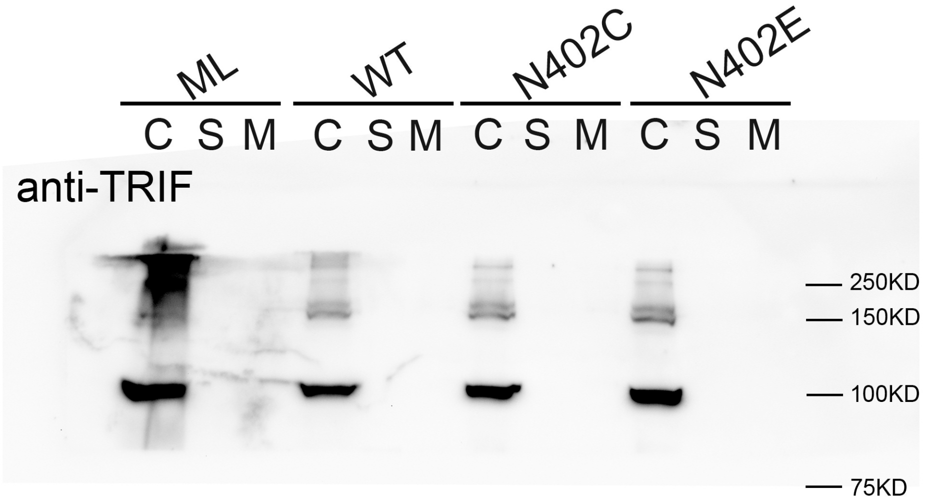

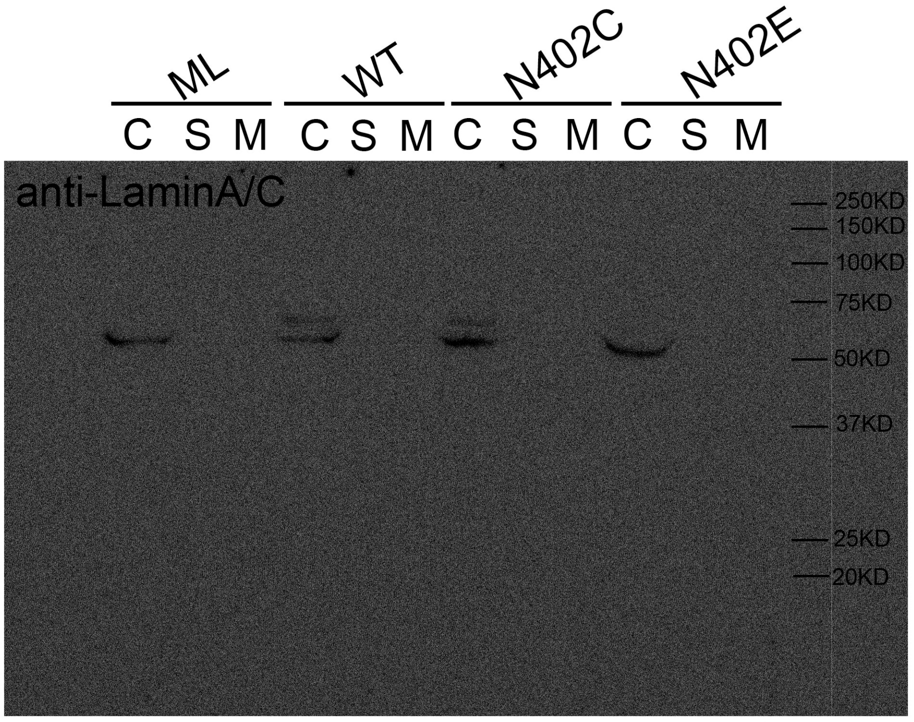

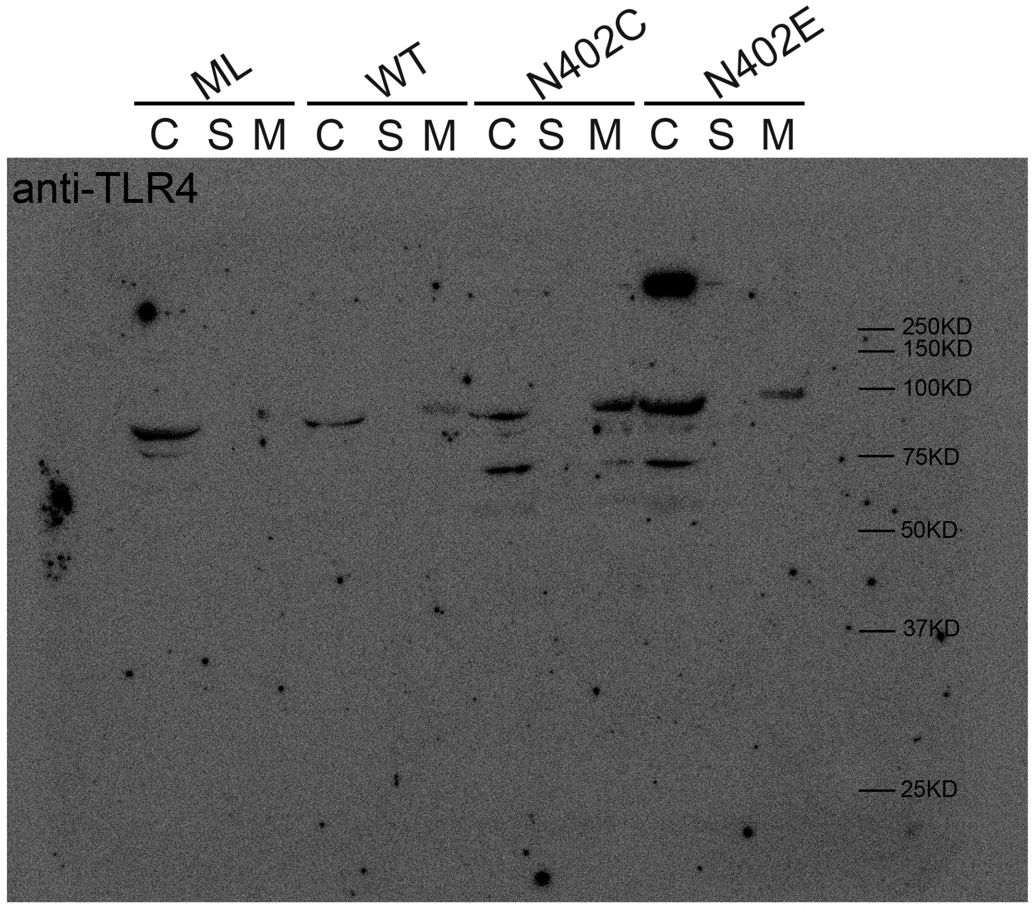

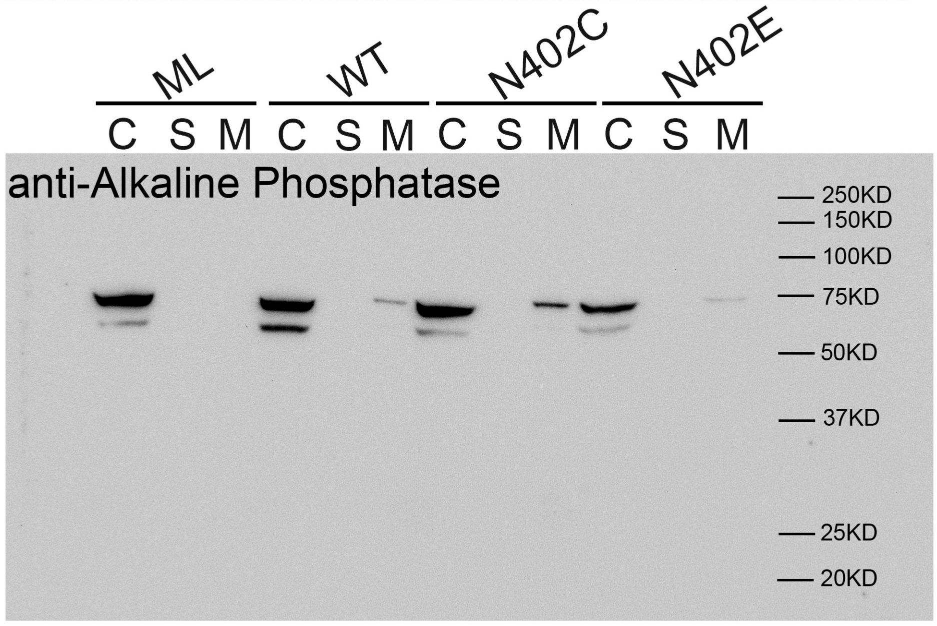

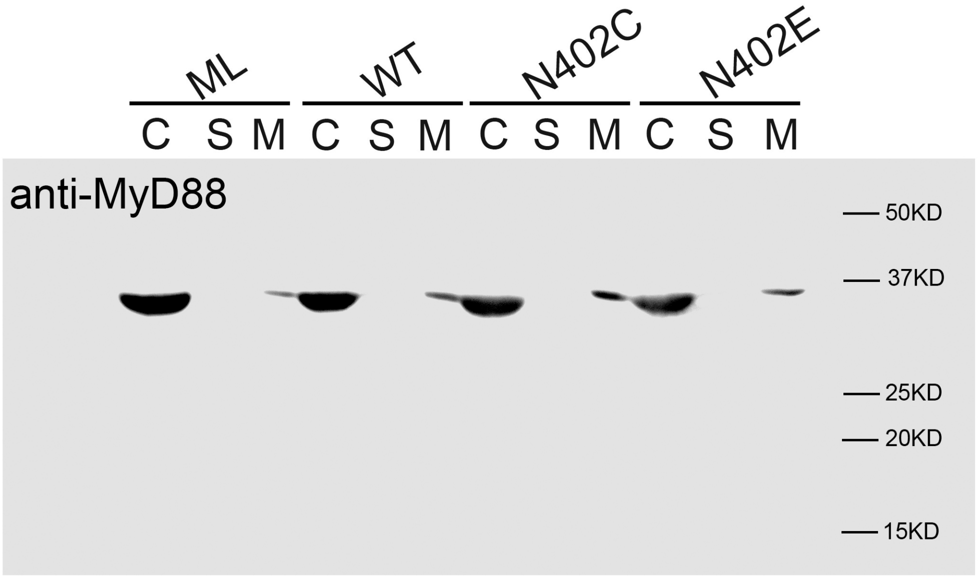

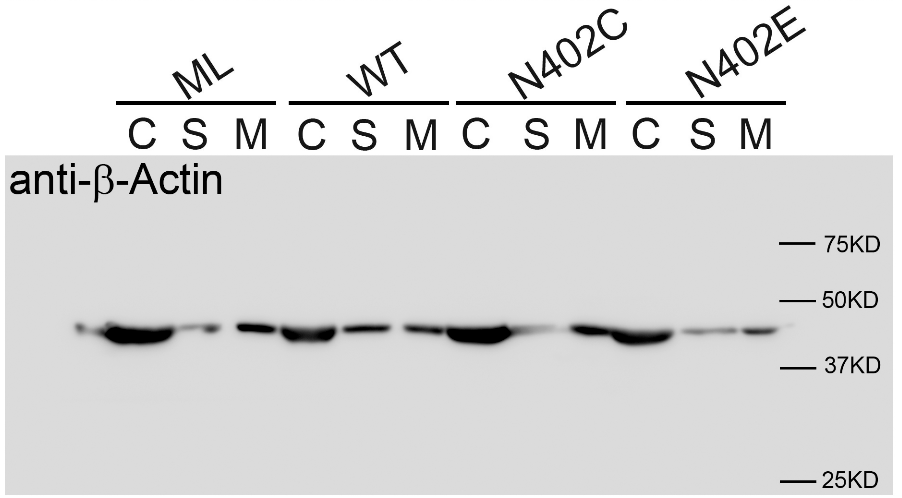

Supplementary Figure S2. Pore formation enhances receptor shedding. The full western blots from Fig 5B are shown. In many cases, the blots were cut following Ponceau S stain and prior to staining with primary antibody. C = cell pellet, S = high speed supernatant, M = microvesicle pellet. Size in kilodaltons (KD) is shown.
